# Methyl-ketones in the scent glands of Opiliones: a chemical trait of cyphophthalmi retrieved in the dyspnoan *Nemastoma triste*

**DOI:** 10.1007/s00049-018-0257-5

**Published:** 2018-04-06

**Authors:** Miriam Schaider, Tone Novak, Christian Komposch, Hans-Jörg Leis, Günther Raspotnig

**Affiliations:** 1 Institute of Biology, University Graz, Universitätsplatz 2, 8010 Graz, Austria; 2 Department of Biology, University of Maribor, Koroška 160, 2000 Maribor, Slovenia; 3 Institute of Animal Ecology and Landscape Planning, ÖKOTEAM, Bergmanngasse 22, 8010 Graz, Austria; 4 Research Unit of Analytical Mass Spectrometry, Cell Biology and Biochemistry of Inborn Errors of Metabolism, Department of Pediatrics, Medical University Graz, Auenbruggerplatz 30, 8036 Graz, Austria

**Keywords:** Nemastomatidae, Scent glands, Methyl-ketones, Naphthoquinones, Sclerosomatid compounds

## Abstract

The homologous and phylogenetically old scent glands of harvestmen—also called defensive or repugnatorial glands—represent an ideal system for a model reconstruction of the evolutionary history of exocrine secretion chemistry (“phylogenetic chemosystematics”). While the secretions of Laniatores (mainly phenols, benzoquinones), Cyphophthalmi (naphthoquinones, chloro-naphthoquinones, methyl-ketones) and some Eupnoi (naphthoquinones, ethyl-ketones) are fairly well studied, one open question refers to the still largely enigmatic scent gland chemistry of representatives of the suborder Dyspnoi and the relation of dyspnoan chemistry to the remaining suborders. We here report on the secretion of a nemastomatid Dyspnoi, *Nemastoma triste,* which is composed of straight-chain methyl-ketones (heptan-2-one, nonan-2-one, 6-tridecen-2-one, 8-tridecen-2-one), methyl-branched methyl-ketones (5-methyl-heptan-2-one, 6-methyl-nonan-2-one), naphthoquinones (1,4-naphthoquinone, 6-methyl-1,4-naphthoquinone) and chloro-naphthoquinones (4-chloro-1,2-naphthoquinone, 4-chloro-6-methyl-1,2-naphthoquinone). Chemically, the secretions of *N. triste* are remarkably reminiscent of those found in Cyphophthalmi. While naphthoquinones are widely distributed across the scent gland secretions of harvestmen (all suborders except Laniatores), methyl-ketones and chloro-naphthoquinones arise as linking elements between cyphophthalmid and dyspnoan scent gland chemistry.

## Introduction

All harvestmen (Opiliones) possess a pair of large, prosomal, sac-like glands, the so-called scent glands. The secretions of these glands are generally considered defensive (e.g., Duffield et al. 1981; Estable et al. 1955; Eisner et al. 2004; Raspotnig et al. 2005) although other functions, such as alarm pheromonal (Machado et al. 2002) and antibiotic activity (Estable et al. 1955), have also been reported. Opilionid scent glands represent an extremely old exocrine system, possibly having been subjected to an evolution of more than 400 million years (Garwood et al. 2011, 2014). As a consequence, scent glands and their secretions underwent many evolutionary modifications that led to the extant diversity of glandular character states in Opiliones, e.g., scent gland openings on prominent tubercles (Raspotnig et al. 2005) versus scent gland openings hidden under integumental folds (Schaider et al. 2010).

Regarding scent gland secretions, the chemical composition of exudates appears to reflect the phyletic lineages among opilionids. In detail, suborders are characterized by the presence of (1) naphthoquinones and methyl-ketones in the Cyphophthalmi (Raspotnig et al. 2005, 2012; Jones et al. 2009); (2) naphthoquinones, anthraquinones, and certain acyclic ketones in the Dyspnoi (Raspotnig et al. 2010, 2014); (3) naphthoquinones and ethyl-ketones in the Eupnoi (Blum and Edgar 1971; Meinwald et al. 1971; Jones et al. 1976, 1977; Wiemer et al. 1978; Ekpa et al. 1985; Raspotnig et al. 2015), and (4) phenols, benzoquinones, terpenes, vinyl-ketones and nitrogen-containing compounds in the Laniatores (e.g., Eisner et al. 1971; Segovia et al. 2015; summarized in Raspotnig 2012).

Remarkably, ketones are present in the scent gland secretions of all four suborders. If these compounds represented products of different character states of one particular biosynthetic pathway originating from a common ancestor of harvestmen, ketones would be ideal to trace the evolutionary history of this class of compounds all across opilionid secretions. However, one so far unresolved issue refers to the still poorly known secretion chemistry in the suborder Dyspnoi which might be considered as a chemical link between the methyl-ketone-rich chemistry of Cyphophthalmi and the various acyclic compounds from sclerosomatid Eupnoi (“sclerosomatid compounds” sensu Raspotnig 2012). Recently, two types of ketones (unsaturated methyl-ketones and saturated ethyl-ketones) were reported from representatives of the nemastomatid genus *Carinostoma* (Raspotnig et al. 2014), but these neither showed identity to the characteristic 4-methyl-substituted ethyl-ketones from Sclerosomatidae nor to the straight-chain methyl-ketones of Cyphophthalmi. We here provide further data on ketone-rich scent gland secretions in Dyspnoi, this time focusing on the secretion of *Nemastoma triste*.

## Materials and methods

**Fig. 1:**
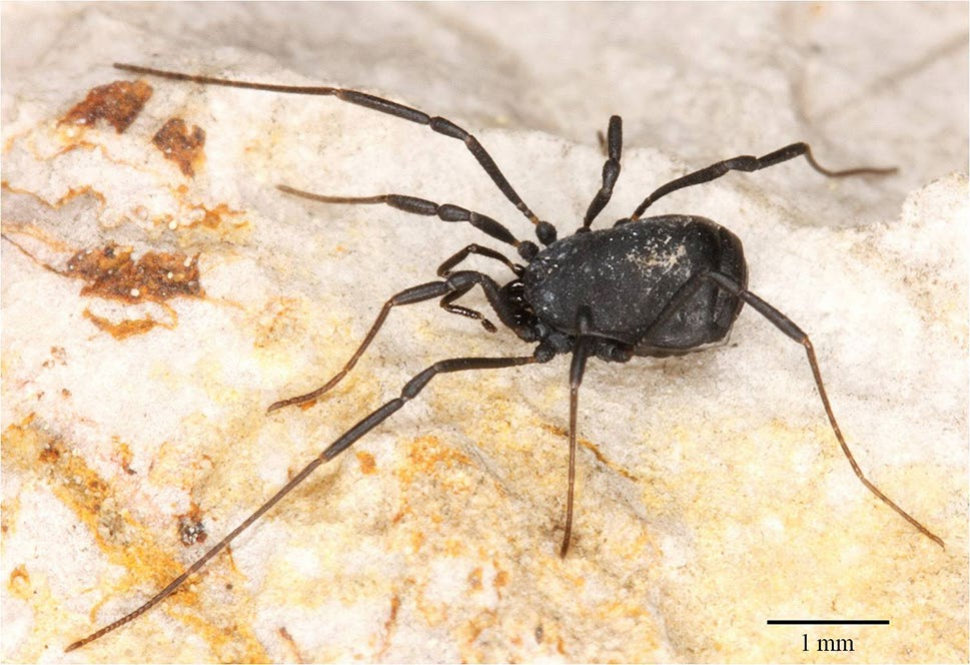
A female of *Nemastoma triste* (from Gesäuse, Styria, Austria)

*Nemastoma triste* (C. L. Koch, 1835) is a small (body length 1.5–1.9 mm) harvestman (Fig. 1), endemic to the Eastern Alps and some Central-European mountains (Martens 1978). Its vertical distribution reaches from the lowlands up to 2380 m in the alpine zone. Habitats are the litter of forests and natural grasslands above the timber line (Komposch and Gruber 2004). Specimens were collected by hand from logs, moos and loose organic matter, or extracted from sieved leaf litter samples between May and September 2012, 2013, and 2017. In total, 142 adult individuals of *N. triste* were collected at different locations in Austria (55) and Slovenia (87) (Table 1). Voucher specimens are deposited at the Institute of Biology (University of Graz) and the Slovenian Museum of Natural History (Ljubljana).

**Table 1:** Collection data: in total, 142 individuals of *Nemastoma triste* were used for this study

Collection date	Locations	Geographic coordinates	No of specimens
05.08.2012	AUT: Styria, Lassing, Blosen	47.517027, 14.272837	1 ♀
14.08.2012	SLO: Dravograd, Mount Košenjak	46.641801, 15.034167	17♀♀,11♂♂
11.09.2012	AUT: Salzburg, Kaprun, Sigmund Thun Klamm	47.257932, 12.737773	1 individual
14.09.2012	AUT: Styria, Präbichl	47.51667, 14.95	1 ♀, 2 ♂♂
14.09.2012	AUT: Styria, Peggau, Lurgrotte	47.201588, 15.353340	1 ♀
14.09.2012	AUT: Styria, Reiteregg, Feistritzhöhe	47.05, 15.266667	1 ♀
07.05.2013	AUT: Styria, Soboth, Schweig	46.721111, 15.083056	3 individuals
08.07.2017	AUT: Styria, Gesäuse, Hartelsgraben	47.566786, 14.697167	1 ♀, 1♂
09.08.2017	SLO: Sv. Urban	46.649798, 15.073275	1♀, 2 ♂♂
23.08.2017	SLO: Rateče, Mount Peč	46.523389, 13.715948	4 ♀♀, 2 ♂♂
23.08.2017	SLO: Jelendol, Mount Gromov	46.422556, 14.357833	7 ♀♀, 15 ♂♂
05.09.2017	SLO: Sadonikhöhe	46.335194, 14.597833	1 ♀, 5 ♂♂
23.09.2017	SLO: Rateče, Mount Peč	46.523389, 13.715944	11 ♀♀, 11 ♂♂
12.10.2017	AUT: Styria, Fischbacher Alpen, Pfaffen	47.557778, 15.796667	10 ♀♀, 2 ♂♂
16.10.2017	AUT: Salzburg, Lungau, Lessach	47.200833, 13.796389	10♀♀, 9 ♂♂
17.10.2017	AUT: Salzburg, Lungau, Sauerfeld	47.122222, 13.885278	7 ♀♀, 5 ♂♂

*AUT* Austria, *SLO* Slovenia

135 specimens were used for the preparation of scent gland extracts. Secretions were obtained by extracting single living individuals in 50 µl hexane (Sigma, Vienna, Austria) for 4 h as previously described for other nemastomatids (Raspotnig et al. 2017). Aliquots of the extracts (1.5 µl) were analyzed by gas chromatography–mass spectrometry (GC/MS), using a Trace gas chromatograph coupled to a DSQ I mass spectrometer, both from Thermo (Vienna, Austria). GC/MS conditions were as previously described (Raspotnig et al. 2017). Retention indices (RI) for detected compounds were calculated using a standard alkane mixture according to Van den Dool and Kratz (1963).

Reference compounds, namely heptan-2-one, nonan-2-one, 6-methyl-heptan-2-one, and 1,4-naphthoquinone were purchased from Sigma (Vienna, Austria), 6-methyl-nonan-2-one from AKos (Steinen, Germany). 6-Methyl-1,4-naphthoquinone was synthesized according to Bruce and Thomson (1952). For the exact determination of the double bond in the tridecen-2-ones, dimethyldisulfide (DMDS) derivatives were prepared by a method modified from Vincenti et al. (1987): 70 µl of pooled extract were mixed with 100 µl hexane, 100 µl of DMDS solution, 20 µl iodine (60 mg iodine in 1 ml ether) and incubated at 75 °C for 1 h. After cooling to room temperature, 200 µl of 5% Na_2_S_2_O_3_ and 500 µl hexane were added and the mixture was rigorously stirred. The upper organic layer was removed and gently reduced under nitrogen. The residue was dissolved in 60 µl hexane and used for GC/MS-analysis. *O*-Methyl oximes were prepared as described in Raspotnig et al. (2014) using methoxamine (MOX) reagent (= 2% methoxyamine-hydrogen chloride in pyridine, Thermo, Vienna, Austria). As a reference for 6-tridecen-2-one and the chloro-naphthoquinones, we used an extract of *Cyphophthalmus duricorius* from which these compounds had already been described (Raspotnig et al. 2005).

Statistics [a multivariate comparison of secretion profiles by non-metric multidimensional scaling (NMDS) as well as a permutational multivariate analysis of variance (PERMANOVA) based on 9999 permutations, both using Bray-Curtis dissimilarity] was performed using PAST (PAlaenotological STatistics, version 3.19).

## Results and discussion

**Fig. 2:**
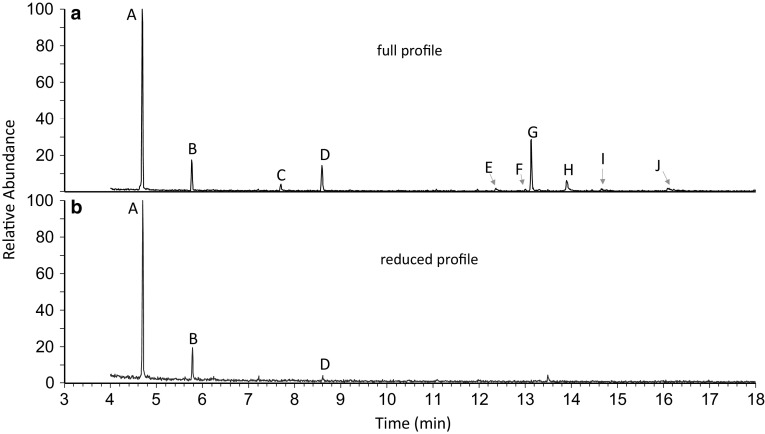
Gas chromatographic profiles of the scent gland secretion of *Nemastoma triste*. **a** “Full profile” of an individual with well-filled glands; **b** “reduced profile” of an individual with partly emptied glands (see text for details). Peak A (heptan-2-one), B (5-methyl-heptan-2-one), C (nonan-2-one), D (6-methyl-nonan-2-one), E (1,4-naphthoquinone), F (6-tridecen-2-one), G (8-tridecen-2-one), H (6-methyl-1,4-naphthoquinone), I (4-chloro-1,2-naphthoquinone), J (4-chloro-6-methyl-1,2-naphthoquinone)

A total of 10 components were found in the scent gland extracts of *Nemastoma triste* (Peaks A–J in Fig. 2 and Table 2). Components A–D showed prominent fragment ions at *m*/*z* 58 arising from McLafferty rearrangement as characteristic for saturated methyl-ketones (= 2-ketones), together with molecular ions at *m/z* 114 (compound A), *m*/*z* 128 (compound B), *m*/*z* 142 (compound C), and *m*/*z* 156 (compound D). This data indicated a series of methyl-ketones from C_7_ to C_10_. However, only compounds A and C appeared to be straight-chain methyl-ketones and were confirmed as heptan-2-one and nonan-2-one by comparison to authentic standards. Component B appeared to be a heptan-2-one with an additional methyl-branch, possibly in position 3, 4, 5 or 6. The mass spectra of 3-methyl-heptan-2-one and 4-methyl-heptan-2-one are characterized by prominent fragment ions at *m*/*z* 72 and *m*/*z* 85, respectively (Linstrom and Mallard, retrieved February 20, 2018), and thus, clearly differed from the mass spectrum of component B. Authentic 6-methyl-heptan-2-one had a similar spectrum, but eluted slightly earlier (RI = 955) than component B (RI = 964) and was thus also excluded, leaving the only possibility of 5-methyl-heptan-2-one open. Both mass spectrum and RI for 5-methyl-heptan-2-one were checked (Linstrom and Mallard, retrieved February 20, 2018; Owens et al. 1997: RI = 971), eventually well matching compound B. Comparably, compound D appeared to be a methyl-branched nonan-2-one, identified as 6-methyl-nonan-2-one by comparison to an authentic standard. Component F (M^+^ at *m*/*z* 196) exhibited the fragmentation pattern of a monounsaturated methyl-ketone, reminiscent of 6-tridecen-2-one and 7-tridecen-2-one from the secretion of *Cyphophthalmus duricorius* (Raspotnig et al. 2005). The retention time of compound F matched 6-tridecen-2-one from a *C. duricorius*-extract (measured as a reference), and its DMDS-adduct (M^+^ at *m*/*z* 290) confirmed the double bond in position 6 (symmetrical cleavage and isobar fragments at *m*/*z* 145). Compound F was accompanied by a shortly later-eluting isomer of larger amount (compound G) which showed mass spectrum indistinguishable from compound F. The DMDS-adduct of G (M^+^ at *m*/*z* 290) exhibited two prominent fragments at *m*/*z* 117 and *m*/*z* 173, indicating the double bond either in position 4 (keto-group in the 117-fragment) or position 8 (keto-group in the 173-fragment). After MOX-derivatization of the DMDS-adduct (M^+^ at *m*/*z* 319), the fragment ion at *m*/*z* 173 shifted to *m*/*z* 202 (plus 29 mass units), proving that this particular part of the molecule carried the keto-group. This pattern is thus consistent with the structure of an 8-tridecen-2-one. Components E, H, I and J were already known from previous studies (Raspotnig et al. 2005, 2017) and readily identified as 1,4-naphthoquinone (M^+^ at *m*/*z* 158), 6-methyl-1,4-naphthoquinone (M^+^ at *m*/*z* 172), 4-chloro-1,2-naphthoquinone (M^+^ at *m*/*z* 192/194), and 4-chloro-6-methyl-1,2-naphthoquinone (M^+^ at *m*/*z* 208/206), respectively.

**Table 2:** Gas chromatographic and mass spectrometric data to the scent gland secretion of *Nemastoma triste*

Peak	RI measured (authentic reference)	Mass spectrometric fragmentation *m*/*z* (relative intensity)	Identified as	Structure	Relative amount^c^
A	892 (891^a^)	**114 (M**^+^, **8)**, 99 (5),85 (5), 72 (7), 71 (25), 58 (96), 43 (100)	Heptan-2-one		40 ± 12
B	964 (971^b^)	**128 (M**^+^, **3)**, 113 (2), 99 (3), 95 (10), 71 (52), 70 (45), 58 (52), 43 (100)	5-Methyl-heptan-2-one		12 ± 4
C	1091 (1092)	**142 (M**^+^, **8)**, 127 (6), 100 (3), 86 (5), 71 (27), 59 (22), 58 (100), 57 (53), 43 (77)	Nonan-2-one		3 ± 1
D	1050 (1051)	**156 (M**^+^, **4)**, 141 (3), 123 (4), 109 (4), 99 (17), 98 (35), 83 (5), 81 (8), 71 (60), 58 (60), 57 (65), 43 (100)	6-Methyl-nonan-2-one		13 ± 5
E	1420 (1419)	160 (M + 2, 15), 159 (M + 1, 13), **158 (M**^+^, **100)**, 131 (12), 130 (37), 104 (43), 102 (54), 76 (34), 57 (24)	1,4-Naphthoquinone		5 ± 4
F	1473 (1473)	**196 (M**^+^, **2)**, 178 (2), 138 (11), 125 (7), 110 (23), 109 (12), 96 (29), 95 (15), 82 (26), 81 (35), 79 (14), 68 (22), 67 (29), 55 (23), 54 (34), 43 (100), 41 (51)	6-Tridecen-2-one		< 1
G	1483	**196 (M**^+^, **2)**, 178 (4), 138 (11), 125 (30), 114 (9), 111 (16), 97 (37), 96 (30), 81 (37), 79 (32), 71 (63), 55 (61), 43 (100)	8-Tridecen-2-one		19 ± 7
H	1544 (1544)	174 (M + 2, 10), 173 (M + 1, 12), **172 (M**^+^, **100)**, 157 (8), 144 (31), 118 (36), 115 (44), 90 (21), 89 (30)	6-Methyl-1,4-naphthoquinone		9 ± 5
I	1605 (1604)	**194**/**192 (M**^+^, **34**/**100)**, 164 (11), 157 (37), 129 (67), 104 (15), 101 (15), 76 (19), 75 (12)	4-Chloro-1,2-naphthoquinone		< 1
J	1734 (1736)	**208**/**206 (M**^+^, **28**/**100)**, 191 (8), 178 (15), 171 (43), 143 (55), 118 (19), 115 (27), 90 (16), 89 (24), 63 (10)	4-Chloro-6-methyl-1,2-naphthoquinone		< 1

The molecular ion (M^+^) is marked in bold. Retention indices (RI) were calculated according to van den Dool and Kratz (1963)

^a^RI as reported by Methven et al. (2007)

^b^RI as reported by Owens et al. (1997)

^c^Given in % of peak area of particular compounds relative to the total area of all secretion compounds. The values refer to “full profiles” (*n* = 28) as described in the text

The detected amounts of secretion considerably varied, most likely according to the filling state of glands at the time of extraction (or to the degree of disturbance of individuals, respectively). While, 21.4% (= 28 individuals) showed large amounts of secretions and hence a full scent gland secretion profile of 10 compounds (including minor compounds), 69.5% (= 91 individuals) exhibited a reduced profile (minor compounds in traces or not detectable any more) (Fig. 2). In 12.2% of the extracts (= 16 individuals) no secretion component was detected at all. Collection and transport of the specimens certainly affected the individuals and probably caused an early emission of parts of the secretion and thus, reduced profiles in our analyses.

**Fig. 3:**
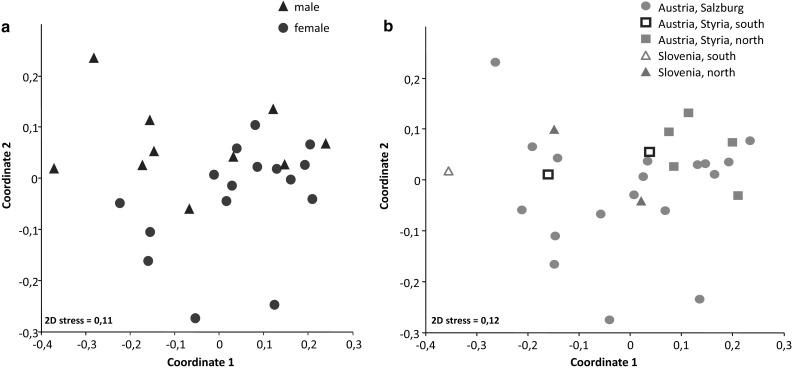
Comparison of individual scent gland secretion-profiles by non-metric multidimensional scaling (NMDS) using the Bray–Curtis index of dissimilarity, indicating homogeneity of profiles. **a** Males and females (26 out of 28 “full” extracts considered; for the remaining 2 extracts, the sex of extracted individuals was not determined); **b** individuals from 5 different populations in Austria and Slovenia (all 28 “full” profiles considered)

Statistics were carried out based on “full” extracts only (*n* = 28). The relation in the composition between male and female extracts was visualized by NMDS (*n* = 26; for 2 extracts, the sex was not determined) (Fig. 3a). For a detailed statistical comparison of male and female profiles, we performed a PERMANOVA, based on 12 females and 6 males from our largest population (Salzburg), showing no difference (pseudo-*F* = 1.028, *p* = 0.386). Comparably, profiles of individuals of different populations exhibited no difference [Fig. 3b; PERMANOVA for Austria vs. Slovenia (*n* = 28): pseudo-*F* = 2.178; *p* = 0.102; among single sub-populations (Salzburg *n* = 18, Styria north *n* = 5, Styria south *n* = 2; Slovenia north *n* = 2): pseudo-*F* = 1.421; *p* = 0.197. The population from “Slovenia south” with *n* = 1 was eliminated from the calculation].

To estimate the absolute amounts of secretion, the amount of heptan-2-one (= main component) was determined for individuals from the Salzburg-population (*n* = 18). Based on synthetic heptan-2-one as an external standard, we found about 230 ng heptan-2-one/individual (calc. 229.8 ± 160.7 ng), reflecting a wide range from 32.5 to 532.5 ng. Interestingly, male extracts (*n* = 6) contained significantly less quantities (calc. 105.6 ± 95.1 ng heptan-2-one/individual) than females (*n* = 12) (calc. 291.9 ± 152.2 ng; two-sample *t* test: *t*_(*df* = 14.902)_ = 3.177, *p* = 0.006). This result may be explained by the slightly smaller body size of males which obviously also affected the stored amount of secretion. To assess the potential maximum filling of glands, the absolute amount of heptan-2-one/individual based on the top 5 of “full” extracts was calculated, indicating that well-filled glands of an individual corresponded to about 440 ng heptan-2-one (calc. 440.2 ± 61.7 ng; max = 532.5 ng). With heptan-2-one amounting for approximately 40% of the secretion (at least on the basis of comparison of peak areas: see Table 2), a single individual with well-filled glands may possess around 1 µg of secretion (calc. 1100.5 ± 154.2 ng; max = 1331.2 ng).

With respect to the evolutionary history of scent gland secretions, dyspnoan scent gland chemistry arises an important key to better understand the evolution of gland chemistry in harvestmen. In *N. triste* the whole composition of the secretion closely resembles the profiles known from cyphophthalmids, i.e., several methyl-ketones are mixed up with naphthoquinones and chloro-naphthoquinones (Raspotnig et al. 2005, 2012; Jones et al. 2009). While methyl-ketones are widespread in arthropod secretions (Blum 1981), these compounds are rare in opilionids and have been considered limited to the scent gland secretions of Cyphophthalmi. By contrast, ethyl-ketones were associated with secretions in Eupnoi and some Laniatores (Raspotnig 2012). The detection of 6-methyl-5-hepten-2-one, an unsaturated methyl-ketone, and octan-3-one, a saturated ethyl-ketone, in the scent gland secretion of the nemastomatids *Carinostoma elegans* and *C. ornatum* (Raspotnig et al. 2014) already indicated that dyspnoan scent gland secretions might represent a chemical link between the suborders. We here provide first evidence for the presence of a markedly methyl-ketone-based secretion apart from Cyphophthalmi, corroborating the intermediate position of dyspnoan scent gland secretions. One ketone compound (6-tridecen-2-one), even though a minor component in *N. triste*, is even shared with cyphophthalmids, suggesting the possibility of a common and ancient pathway of methyl-ketone formation. Though clearly belonging to the class of methyl-ketones as well, the remaining five ketones detected in our study (heptan-2-one, nonan-2-one, 5-methyl-heptan-2-one, 6-methyl-nonan-2-one, and 8-tridecen-2-one) are new compounds for opilionid secretions. However, while most of the ketones in *N. triste* are short-chained and highly volatile, methyl-ketones in the Cyphophthalmi generally represent rather long-chained molecules (mainly C_13_- and C_15_-methyl-ketones). *Nemastoma triste* may thus be classified with the “volatile type” of dyspnoan scents gland secretions as already described for species of *Carinostoma* (Raspotnig et al. 2014). Comparably, chloro-naphthoquinones are rare components in land animals, having been considered restricted to cyphophthalmids but just recently recovered in Nemastomatinae (Raspotnig et al. 2005, 2012, 2017). Even though nothing is known about their biosynthesis/formation process in scent glands, these compounds might also represent a chemical synapomorphy of *N. triste* and other species of *Nemastoma* (Raspotnig et al. 2017) with Cyphophthalmi. *Nemastoma triste* is closely related to *N. schuelleri* (Komposch and Gruber 2004); thus it would be worthwhile to investigate the chemistry of the latter species as well.

The scent glands of many dyspnoans tend to diverge from the general organization of defensive glands in harvestmen, showing aberrant morphological features together with not easily dischargeable/detectable secretion in many species, even casting doubt on their defensive role (Schaider and Raspotnig 2009; Schaider et al. 2010, 2011; Raspotnig et al. 2014). For *N. triste* we assume that the methyl-ketones and naphthoquinones are used as allomones, just as scent gland secretions in most other Opiliones. Methyl-ketones in arthropods may show a variety of effects, such as alarm pheromonal activity in ants and other hymenopterans (Blum 1981; Cheng et al. 2017) or sex pheromonal functions in Trichoptera (Löfstedt et al. 2008). A pheromonal function for *N. triste*, however, has not yet been demonstrated.
